# Following of aging process in a new motor skill learning model, “pot jumping” in rats

**DOI:** 10.1007/s11357-019-00073-3

**Published:** 2019-05-25

**Authors:** Aliz Judit Ernyey, Tiago Grohmann Pereira, Kata Kozma, Shima Kouhnavardi, Ferenc Kassai, István Gyertyán

**Affiliations:** MTA-SE NAP B Cognitive Translational Behavioural Pharmacology Group, Department of Pharmacology and Pharmacotherapy, Faculty of Medicine, Semmelweis University, Nagyvárad tér 4, Budapest, H-1089 Hungary

**Keywords:** Procedural learning and memory, Voluntary motor task, Age-related motor impairment motivation

## Abstract

Impairment of procedural memory is a frequent and severe symptom in many neurological and psychiatric diseases as well as during aging. Our aim was to establish an assay in rats in which procedural learning and changes in performance can be studied on the long term. The work was done in the frame of a larger project aiming to establish a complex cognitive animal test battery of high translational value. The equipment was a 190-cm-diameter circular water tank where 12 flower pots were placed upside down in a circle with increasing distances (18–46 cm) between the adjacent ones. Male Lister Hooded and Long-Evans rats were allowed to move on the pots for 3 min. The arena was filled with shallow water to make the rats stay on the pots. Animals were obviously motivated to move around on the pots; however, the distance which required jumping (> 26 cm) meant a barrier for some of them. Development of motor skill was measured by the longest distance successfully spanned. A relatively flat bell-shaped age dependence was observed, with a peak at 13 months of age. A gradual decline in performance could be observed after the age of 20 months which preceded the appearance of overt physical weakness. Long-Evans rats showed more homogeneous performance and higher individual stability than Lister Hooded rats. The method is appropriate to study the development of motor learning and to follow its age-dependent changes. It may also serve as an assay for testing potential drugs for improving motor skills and/or procedural memory.

**Electronic supplementary material:**

The online version of this article (10.1007/s11357-019-00073-3) contains supplementary material, which is available to authorized users.

## Introduction

The pharmacological treatment of age-related cognitive disorders is currently unsatisfactory. Despite the abundance of cognitive enhancer mechanisms identified in basic research, several dozens of pre-clinically promising compounds failed in clinical studies. A major reason may be that the so-called gold-standard animal assays used in fundamental research fail to predict clinical efficacy against complex and robust cognitive defects. The aim of our group has been to establish a rodent cognitive test battery with improved predictive power for the clinical efficacy of potential cognitive enhancers (Gyertyan 2017). The system consists of learning paradigms that model the various human cognitive domains defective in psychiatric and neurodegenerative disorders (Millan et al. 2012). One of these is procedural learning and memory, which is impaired in several age-related diseases, most prominently in Parkinson’s disease (Kim et al. 2018; Belghali et al. 2017), but also weakens during natural aging (Voelcker-Rehage 2008). Our objective was to find a translationally valuable rodent model of procedural learning and memory, which is also suitable for longitudinal follow-up of the performance.

Initially, we applied the widely used rotarod apparatus for measuring motor skill development and motor coordination in rats (Carter et al. 2001; Buitrago et al. 2004). However, this did not provide adequate results with our animals since, during a few repetitive training sessions, they used the apparatus as a “playground”: they frequently jumped down from the rotating rod on purpose, or clung to the separating circular plates and used it as a descending elevator, even though the principle of the assay would be to measure the retention time until falling due to motor impairment. Our rats’ brave and much self-confident behavior could be explained by the intensive handling, habituation to the lab environment, and regular training in other cognitive tasks what we all applied to reduce their stress level and make them fit for the performance requirements.

In searching for an appropriate motor learning assay, we developed the so-called pot jumping test, introduced in this paper. In this paradigm, rats are allowed to freely move on the top of flower pots placed upside down in a circle form in an open arena. The distances between the adjacent pots gradually increase anticlockwise. The arena is filled with shallow water to force the rats to refrain from descending from the pots. This novel task turned out to have several advantages. First, the motor ability can be graded since the alteration of the distances requires continuous adjustment: both too short and too long jumps lead to errors thus falling/slipping into the water. Second, the task enables voluntary exercise; rats can decide individually the longest distance they span between two pots depending on their actual motor and/or mental condition. Thus, the test is sensitive to the motivational state of the animals. Third, during repeated testing, rats can train themselves in mastering the task, namely to move around in the arena. Fourth, the voluntary nature of the task extends the individual variation among the subjects, which may be utilized in detecting differences in learning capabilities. Since motor performance may be enhanced by increasing the motivation of the subjects (Mosberger et al. 2016), we also planned to place food pellets on the pots as rewards for the successful jumps. However, it turned out later that the exploratory drive of the animals is enough for active participation in the task.

After introducing the assay, we also intended to follow the effect of aging on the acquired motor skill. Thus, in the current article, we present the performance of Long-Evans (LE) and Lister Hooded (LH) rats across their lifespan. This longitudinal study has spread out for almost 3 years; therefore, parallel with measuring the age-related performance, we were continuously refining the method until the final pot pattern was set up.

## Materials and methods

### Subjects

Subjects of the study were 36 male Lister Hooded (LH; Charles River, Italy) and 36 male Long-Evans (LE; Janvier, France) rats 7 months and 4 months old, respectively, at the beginning of the experiment. The LH animals were studied until their age of 27 months, when the remaining 27 rats were euthanized for another study. The LE rats were able to participate in the experiment until their age of 38 months, when 11 of them were still alive. Body weight fell in the range of 376–508 g and 385–498 g in case of Lister Hooded rats, and 290–381 g and 283–414 g in case of Long-Evans rats at the beginning and at the end of the test, respectively. Animals were housed in groups of three in 1500-cm^2^ polycarbonate cages with paper tube and wooden chewing bricks as enrichment tools and were regularly exposed to handling throughout the measurements. They were kept on reversed light-dark cycle (dark phase from 4:00 am until 4:00 pm) and restricted food access (commercial pellet rat feed R/M-Z+H produced by SSniff Spezialdiäten GmbH). The amount of food was 45 g for 3 rats supplied at the end of the dark phase, at 3:30 pm. Water was available ad libitum. Parallel to the pot jumping training, the animals participated in various other cognitive tasks. Half of the Long-Evans rats were treated by a putative anti-aging compound from their age of 27 months until death. The treatment did not affect the “pot jumping” performance of the animals; thus, the data of control and treated groups were pooled during the whole experiment period.

The experiments were authorized by the regional animal health authority in Hungary (resolution number PEI/001/3572-4/2014) and conformed to the Hungarian welfare legislation and the EU 63/2010 Directive.

### Apparatus and training procedure

**Fig. 1:**
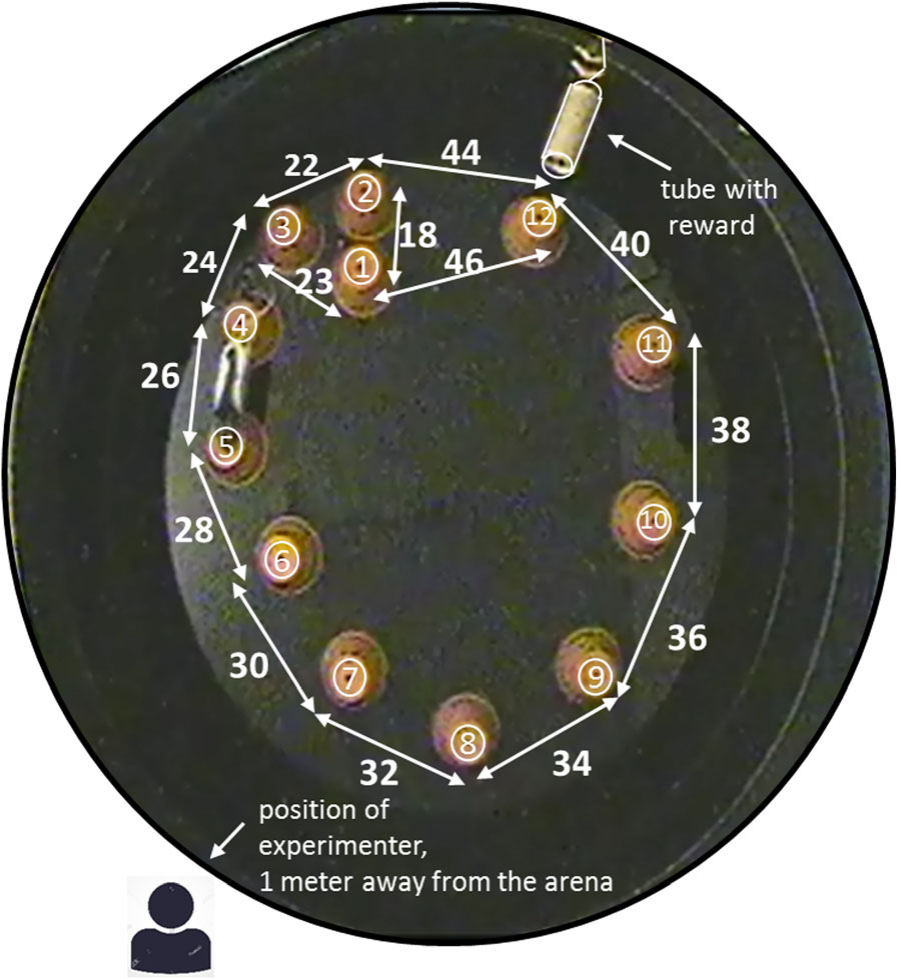
Final pot setup for the training procedure of Long-Evans and Lister Hooded rats

**Fig. 2:**
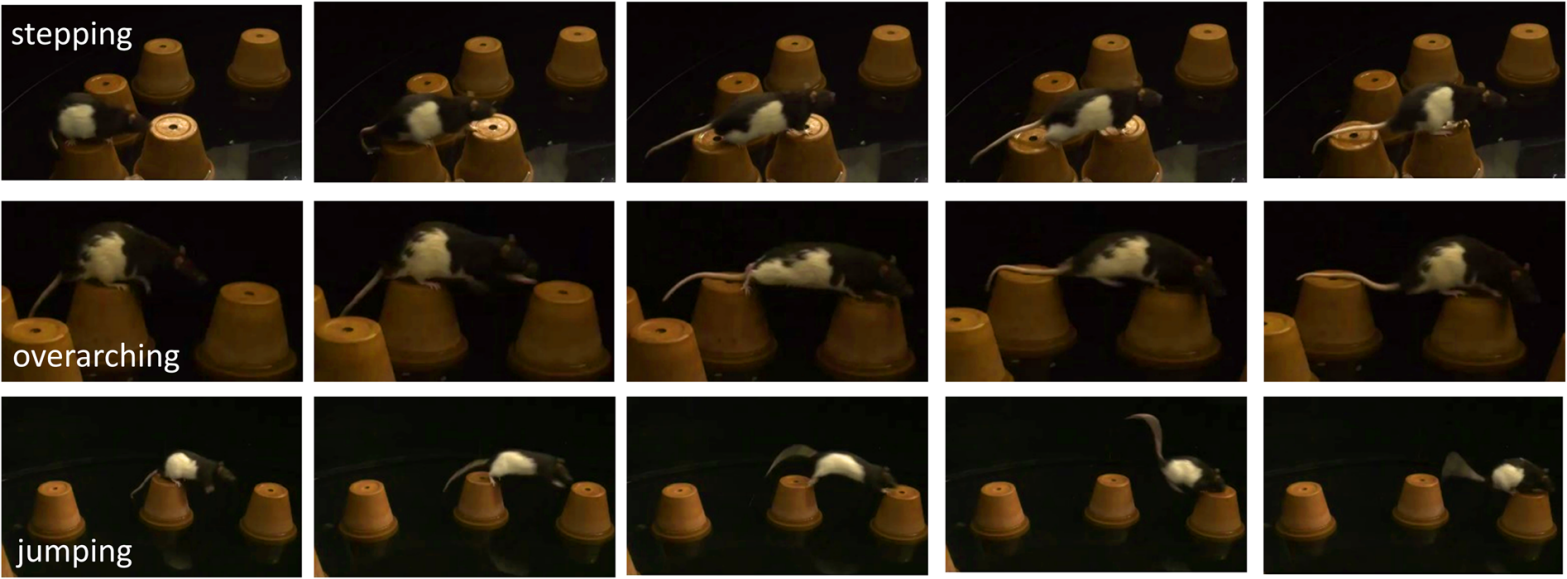
“Stepping,” “overarching,” and “jumping” between two pots by the rat in the test arena

The equipment was a 190-cm-diameter circular open arena with 60-cm-high walls where 12 flower pots (16 cm high, 10 and 17.5 cm wide at base and top, respectively) were placed upside down in a circle form with increasing distances (18–46 cm) between the centers of two adjacent pots (Fig. 1). A horizontally placed paper tube (20 cm long, 8-cm diameter) was suspended above pot 12 on the wall of the arena, so that the animal could climb and hide inside, where one piece of peanut reward could be obtained. Once the animal was placed onto the first pot, the experimenter always returned to the same position—in the vicinity of pot 7—and he/she stayed there until the trial was completed. The arena was filled 6 cm deep with cold water to make the rats refrain from descending from the pots. During a trial, the subject was placed onto pot 1 (the one within the shortest distance (18 cm) to the next) and allowed to move on the pots for 3 min. Regarding motor skill performance, three types of moving between two pots were distinguished: “Stepping”: first, fore- and hind leg of the same side moved forward in succession then fore- and hind leg of the other side followed; “overarching”: two forelegs leaned on the pot ahead while two rear legs were still on the pot behind; “jumping”: all four legs in the air at the same time (Fig. 2). Rats were able to step over the shortest (18 cm) distance; spanning of distances up to 26 cm was possible by “overarching,” while spanning longer than 26 cm was performed by “jumping.” In case the rats fell into water after jumping, it was not considered as successful spanning of the pots. Training sessions were held at least once a month. The experiment was carried out at about 50-lx luminance intensity. Movements of the animals were video-recorded using Smart v3.0 video tracking system software (Panlab, Spain). Motor skill performance was characterized by the longest distance successfully spanned by the rats in a trial.

Fecal boli were removed from the water and from the pots after each trial. Water was exchanged, and the pool was thoroughly cleaned after every week of testing.

### Experimental design

As the method was under development, the training procedure of the rats was continuously adjusted during the experimental period according to the performance of the animals (see Table 1). We started to set up the experimental design using Long-Evans (LE) rats of 4.5 months of age. They were habituated to the circular arena equipped with 12 pots in circle form, 18 cm between them except for pot 12 and 1 that were 24 cm distant from each other. In their age of 5 months, the distances were increased to 24 cm between pots 1 to 6 and 26 cm between pots 6–12. The longest distance to span was 40 cm. Afterwards, the distances on the first and second halves of the circle were increased to 26 cm and 30 cm, respectively. As a next step, from their age of 13 months, LE rats were allowed to jump from 26 to 40 cm, with 2 cm increasing distances between the adjacent pots. Lister Hooded rats started their training with this setup at their age of 7 months. Subsequently, the longest distance was extended to 44 cm (Table 1). The rats performed the test from their age of 21 months (LE) and from age of 14 months (LH) with the final setup shown in Fig. 1.

**Table 1:** Pot arrangements for the training procedure of Long-Evans and Lister Hooded rats. Changes from the previous setup are italicized

Long-Evans rats		Pot number	Between pot distance (cm)	Lister Hooded rats	
Age (months)	Number of training days			Age (months)	Number of training days
4, 5	1	1,2, …12	18		
		12–1:	24		
5–6	2	1 to 6	24		
		6 to 12	*26*		
		12–1	*40*		
10	1	1 to 6	26		
		6 to 12	*30*		
		12–1	*40*		
13–15	5	1–2	26	7–12	15
		2–3	28		
		3–4–5	30		
		5–6–7	32		
		7–8–*9*	34		
		*9–10–11*	36		
		*11–12*	38		
		12–1	40		
19–21	6	1–2	26	13	4
		2–3	28		
		3–4–5	30		
		5–6–7	32		
		7–*8*	34		
		*8–10*	36		
		*10–11*	38		
		*11–12*	40		
		12–1	*44*		
21–38	22	*1 to 12*	*18 to 46* (see Fig. 1)	14–27	15

### Statistical analysis

The mean of the longest distance spanned as a function of the age of the animals was analyzed with repeated measures ANOVA followed by Duncan post hoc tests. The statistical analysis was only performed for the period of 19–27 months (until the termination of LH animals) as the pot setup was mainly the same in this period for both strains. Performance at younger ages was not statistically compared because the two strains were trained on different pot setups and with dissimilar intensity. Moreover, LH rats started the task at an older age (7 months) than LE rats (4.5 months). Missing data due to interim deaths were replaced by the sample mean. Histograms of the individual lifetime longest distance achieved (“personal best”) and also of the most frequently reached longest distance (“most often”) values were plotted, and normal distribution curve was fitted (Origin 2015 software). To compare the proportion of “not leaving” rats (that did not leave the first three pots, i.e., that reached pots until maximum 23-cm distance, but were not able to overarch 24 cm) as well as of “jumper” rats (that were able to jump over at least a distance of 28 cm) between the two strains, *χ*^2^ test was used.

## Results

Moving on the pots required three different types of motor skills: stepping, overarching, or jumping (Fig. 2). The 18-cm distance was simply stepped over by the rats, while intervals up to 26 cm were typically overarched. Greater distances (≥ 28 cm) could be spanned only by jumping; however, it could not be performed by some of the rats (“non-jumpers”). Even for “jumpers,” a certain distance apparently meant a barrier in a given trial. After a more or less continuous sequence of jumps on increasing distances, they stopped on a certain pot and after a variable period of hesitation, they refrained from jumping further and instead, they jumped backwards and returned to the first pots. However, at the next occasion—typically in the learning phase of the task—they often jumped at a longer distance and stopped one or two pots further.

### Age-dependent performance (longest distance spanned)

We followed the performance of the rats across their lifespan, until the termination of LH rats and until the LE rats were physically able to move on the pots. The number of the animals reduced during the experiment as some of them died naturally. Rats that became paralyzed or had bumblefoot (ulcerative pododermatitis) in their old age did not participate in the experiment since they were physically unable to overarch the pots.

**Fig. 3:**
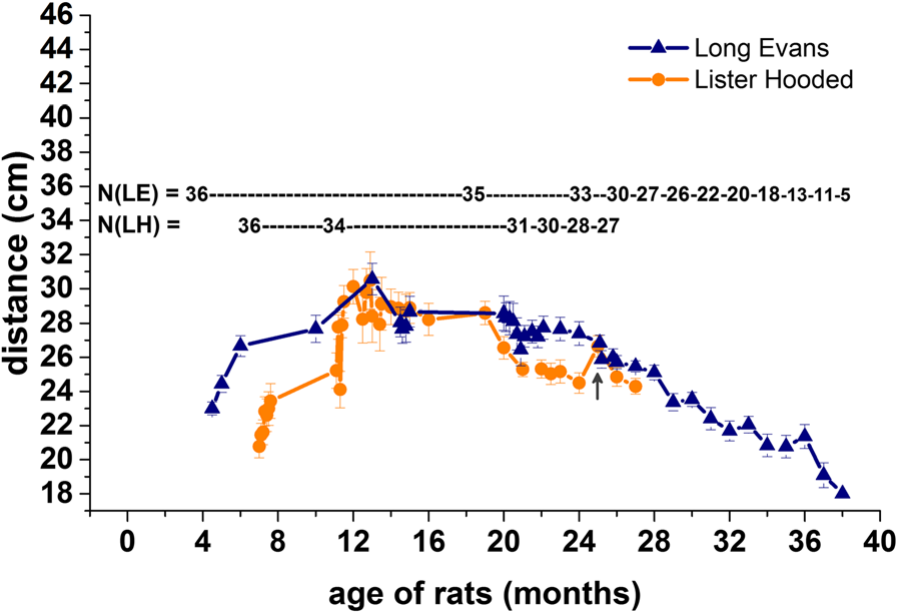
Mean value ± SEM of longest spanned distance of rats depending on their age. N indicates the number of animals participating in the experiment at the given age. Orange (dark gray) circle symbols represent the performance of Lister Hooded (LH) rats and black triangle symbols represent the performance of the Long-Evans (LE) group. For the outlier data point indicated with arrow, *see* “Discussion”

A relatively flat bell-shaped age dependence was observed (Fig. 3); the mean longest distance peaked at the age of 13 months both in LE and LH rats (32.8 and 37.0 cm, respectively). LH rats’ performance started to decrease at a younger age than that of LE rats. Repeated measures ANOVA revealed a significant decrease in performance by time in each strain (“months” effect *F*(9,630) = 13.3, *p* < 0.001); with the LH rats showing a greater decline in the period of 21 to 24 months (“strain” effect (1,70) = 6.1, *p* < 0.05; “months × strain” interaction: *F*(9,630) = 3.2, *p* < 0.001) (Fig. 3).

### “Personal best”

**Fig. 4:**
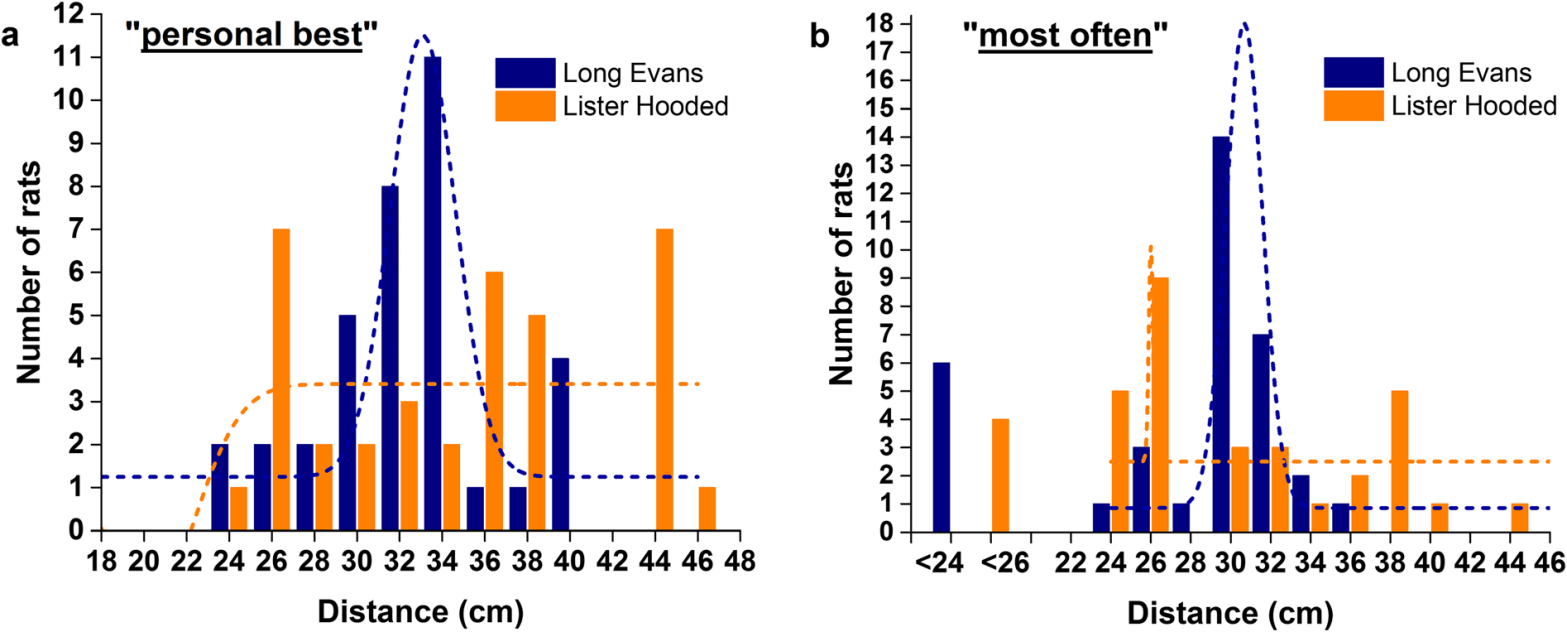
**a** Histograms with Gaussian curve fitting of the “personal best” results of LE and of LH rats across the training period. Blue (black) bars represent the performance of LE rats and orange (gray) bars that of the LH rats. **b** Histograms with Gaussian curve fitting of the most often achieved distances between 10 and 24 months (LE) and 11 and 21 months (LH) age

All of the rats succeeded to span at least 24-cm distance at least once in their life span. The distribution of the “personal best” values of LE animals showed a more or less Gaussian curve between distances of 24 and 40 cm with a mode of 34 cm (11 rats). In contrast, the distribution of LH rats was rather uniform spreading from 24 to 46 cm. There were 4 LE and 8 LH animals which did not move longer than 26 cm (i.e., they did not jump). The “record” of LE rats was 40 cm jumped by 4 animals, while 7 LH rats could reach the 44 cm range, and the “recorder” jumped even 46 cm (Fig. 4a).

### Typical individual performance (distance most often spanned)

We determined for each animal which was the longest distance most often spanned in the period of their age of 10–24 months (LE) and 11–21 months (LH), i.e., in the time interval after the learning phase and before the physical condition-related decline in performance (Fig. 4b). From the fitting of normal distribution curves, 7 LE and 5 LH rats were excluded. These animals typically did not leave the first pot, probably because the shortest possible distance (26 cm) in the abovementioned life period was too long for them to start. LE but not LH distribution fits well to the normal curve. The peak of the fitted Gaussian curve of LE rats was at 30 cm which was jumped by 14 animals as the most often spanned distance. The curve for LH rats peaked at 26 cm by 9 animals. The LE group involved less animals with short as well as with long distances than the LH group. The performance of the LH group was more evenly and widely distributed than that of LE group (Fig. 4b).

### Proportion of the most often spanned distance to all occasions

**Fig. 5:**
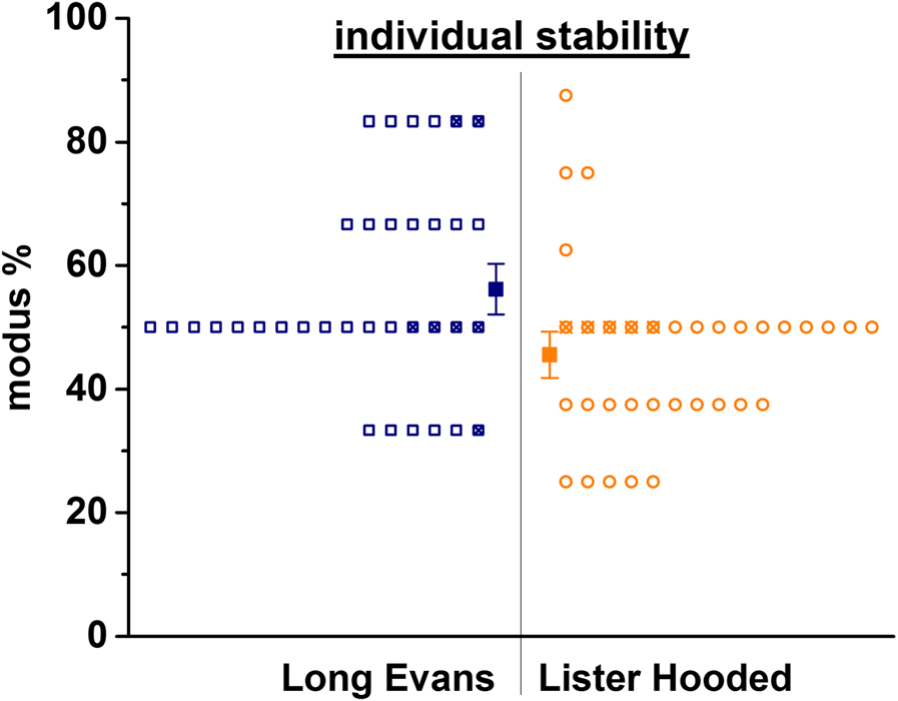
Modi of the individual results between 10 and 24 months (LE) and 11 and 21 months (LH) age. Blue (black) data points show the modi of LE group and orange (gray) data points those of the LH rats. Filled squares represent the mean ± SEM of the values; crossed symbols show the individuals which typically did not leave the first three pots during their life span

As a measure of the stability of individual performances, we calculated the percentage of the number of most often spanned distances relative to all occasions, that is, the mode of the intraindividual performances. One hundred percent would mean the animal reached the same distance in all trials (i.e., highly stable performance) whereas a low percentage indicates variable (unstable) performance. The mean of modi of LE rats (56.2 ± 2.7%) is higher than that of LH rats (45.6 ± 2.5%) indicating a more homogeneous performance in the former strain. The modus of modi describes the value which is characteristic (typical) for most animals: it is uniformly 50% for both strains (Fig. 5).

### Number of rats “not leaving”

**Fig. 6:**
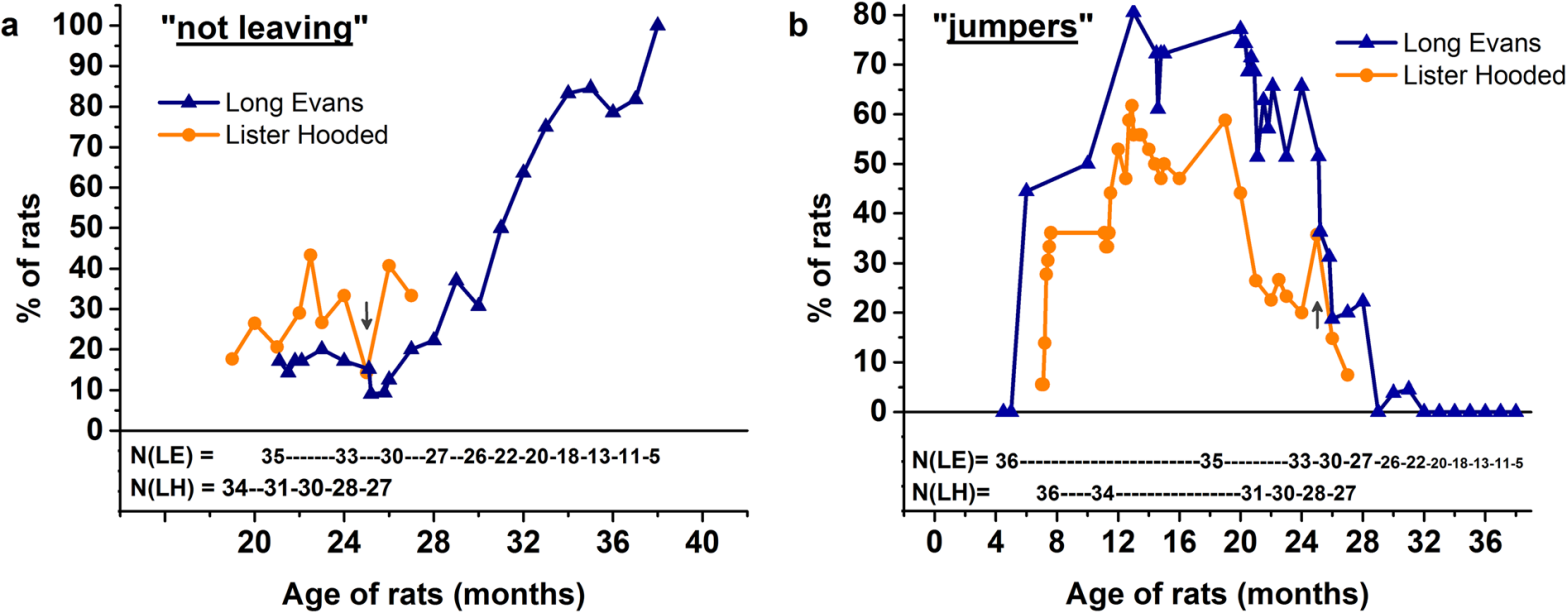
**a** Proportion of “not leaving” rats that spanned maximum 23 cm related to all rats participating in the experiment at the given age. Orange (dark gray) circle symbols and lines represent the performance of Lister Hooded (LH) rats and blue (black) triangle symbols and lines represent the performance of the Long-Evans (LE) group. **b** Proportion of rats spanning distance by jumping related to all rats participating in the experiment at the given age. For the outlier data point indicated with arrow, *see* “Discussion”

The proportion of “not leaving” rats, relative to all rats involved in the experiment at the given age, was plotted from age of 19 months of LH rats, when the decline started. For LE rats, the “not leaving” value is shown only from the age of 21 months, as the pot setup included the 18-22-23 cm triangle form only from that age (Fig. 6a). In the case of LE rats, this value was about 15–20% until the age of 26 months; thereafter, it increased almost continuously until the end of the experimental period. The proportion of “not leaving” rats in LE and LH groups was significantly different in the decline phase between the age of 21 and 24 months (5–5 measurements) (*χ*^2^_(df = 1)_ = 4.46, *p* < 0.05).

### Number of “jumper rats”

The proportion of rats that were able to jump shows a bell-shaped curve in the function of age (Fig. 6b). The number of jumping animals increased until the age of 13 months, remained relatively stable until the age of 19–20 months; thereafter, it started to decrease in both strains. The proportion of jumpers in the LE group was significantly higher than in the LH group between the age of 21 and 24 months (5–5 measurement points, *χ*^2^_(df = 1)_ = 16.75, *p* < 0.001). There was no Long-Evans rat at all that managed to jump over the pots from the age of 32 months (Fig. 6b).

## Discussion

In this newly established motor skill test, animals were allowed to move freely back and forth on the pots which—due to the varying distances between them—continuously required an exact adjusting of the movement in both directions. At the very beginning, we placed reward pellets on the top of each pot to motivate the rats to move over. However, it quickly turned out that rats were obviously motivated to spontaneously move around on the pots without rewarding. Exploration was probably the major driving force in the pot jumping test carried out in a big open arena, despite the fact that rats performed the test in a familiar environment. Thus, only the last pot (i.e., the longest possible distance to jump) was rewarded where peanut was placed into the paper tube, which itself served as a stimulus calling for exploration (Fig. 1). These aspects of the paradigm show similarity to the ladder rung walking test described by Metz and Whishaw (2002). In that assay, rats had to walk on metal rungs that were placed in an irregular pattern with altering distances between them. Also, continuous adjusting was required, although in walking and not jumping. Similarly to our observations, rats were motivated to participate in the task without rewarding.

The gradually increasing distances helped the rats to perfect their movements during progressing, but even “jumpers” definitely stopped at a certain distance despite the fact that the “interpot” distances increased only by 2-cm steps. At this point, they were seemingly hesitating for quite a while between jumping and not jumping—as if they had performed a kind of mental practice—“the imagined rehearsal of a motor act with the specific intent of learning or improving that act” as defined by Mulder et al. (2004)—but then, they refrained from jumping further. However, in the mastering phase of the task, at the next occasion, they usually jumped over the previously only mentally practiced distance and repeated the above process at a longer distance. This trial to trial improvement is in accordance with the observations that motor learning process consists of stages and repetitive training sessions with breaks or sleep in between are more effective than continuous skill learning (Luft and Buitrago (2005). Improved performance in a retest trial was also found in older people studied in a gait-slip task (Liu et al. 2017).

At the beginning of the test period, rats spanned below 28 cm by overarching; however, after gaining some experience, they spanned these distances also by jumping, especially through decreasing distances on the way back to the first pot—obviously because, when rats are in continuous moving on the pots with a given momentum, keeping on jumping requires less physical effort than changing to overarching. A similar mastering in movement technique was observed on the rotarod where trained rats changed their gait patterns from “slow stepping” or “uncoordinated jumping” to “running,” the latter representing a smooth movement sequence with less adjustments necessary to perform the task (Buitrago et al. 2004).

During the test period, motor skill developed to the point that the best performer LH rats jumped over their longest distance directly from pot 1 already at the beginning of the trial (see video in Online Resource 1 and 2). The best performers even kept on jumping after reaching the tube, apparently just for the sake of jumping despite the fact that, sometimes, their attempts led to falling into the water. In contrast, for some of the rats—that were not leaving the triangle of the first three pots—24 cm meant a barrier. Between 20 and 24 months of age, when the decline in performance already began, the proportion of “not leaving” LH animals was about the double of that in LE rats. However, this incapability may rather have been related to anxiety than to an impaired physical condition. At the beginning of the experiment, LE rats had the opportunity to span 24 cm as the shortest distance (4.5 to 6 months of age); however, LH rats’ shortest possible distance to span was 26 cm during their learning period (7 to 11 months of age). Thus, LH rats could not “practice” on shorter distances; probably, that is why they were not able to gain courage to overarch distances above 26 cm.

LE rats showed more homogenous performance than LH rats in all the recorded parameters, i.e. „personal best” values, typical personal performance (distance most often spanned) and individual stability, i.e. proportion of the most often spanned distance to all occasions. The modified pot setup for the two groups may have led to the contrast of the results: LE rats were already older (19 months) than LH rats (13 months), when they had the opportunity to span 44 cm or 46 cm. In their younger age the longest possible distance was 40 cm so they could not try the longer distances; by the age when they could, they probably lost the motivation or courage to jump longer.

Concerning the age-related performance, the mean values of longest distance spanned and the proportion of “jumper” rats improved for both strains on average until their age of 13 months (Fig. 3, Fig. 6b), which period was considered as the phase of mastering the task. The improvement may be explained by an increasing motivation to move around the pots, possibly also owing to the familiar environment by the time. Afterwards, a more or less constant performance followed until the age of 19–20 months. The decline phase started at age of 20 months in LH and 21 months in LE rats, which can be followed through decreasing of the longest spanned distance (Fig. 3), and of the proportion of “jumper” rats (Fig. 6b). Proportion of LE rats not leaving the first three pots started to increase rapidly at the age of 27 months (Fig. 6a). For LH rats, the same time dependence could not be established due to their early euthanasia; however, signs of a starting decline were observed already at age of 22 months. LH rats’ earlier beginning of decline cannot be accounted for body size difference between the two strains as the weights of LH rats were on average higher than that of LE rats during the whole experimental period. The well-preserved physical activity until the age of 19–20 months may have also been backed by the limited food-access regime known to enhance life span and physical fitness (Means et al. 1993; Masoro 2009). The decline in motor performance started thereafter, which might be connected to emerging movement difficulties and/or lack of motivation.

Pot jumping performance of rats across the lifespan may match the human age-related changes in physical activities including motor learning (Voelcker-Rehage 2008). Despite the diverse maximum life expectancy, the shape of life span curves of humans and various model organisms (mice, monkeys, even worms) shows similarity (Mitchell et al. 2015). The rats’ highest achievement at around 1 year of age is consistent to the flourishing physical activity of an average human young adult population about 25–30 years of age whereas the slowly descending performance observed from 19 to 20 months parallels the decreasing performance of people from around 50–60 years.

Behavioral studies that continuously follow the motor function of rodents are scarce in the literature. Jänicke et al. (1983) observed deterioration from age of 20 months in climbing test and chimney test. Impairment of coordination in rotarod test and reaction time in tilting plane test became prominent after 24 months of age while no difference was recognized in spontaneous activity and swimming performance over the monitored life span (4–32 months). Gage et al. (1984) reported that rats at 2 years of age were impaired in swimming, open-field activity, motor coordination assays, and spatial navigation; however, they did not follow the performance over the lifespan. At the same age, rats showed impaired ladder walking ability in the study of Metz and Whishaw (2002). Shukitt-Hale et al. (1998) found impairment in motor performance in several assays (rod walk, wire suspension, plank walk, inclined screen, and accelerating rotarod) already from the age of 12 months in Fischer 344 rats. Lamberty and Gower (1990) also observed age-related decline in activity in open field, hole board, Y maze, and plus maze tests in mice of four different age groups. Fahlström et al. (2011) showed that explorative activity decreased with advancing age in elevated plus maze, open field, and object recognition tasks performed by mice. In a more recent study, age-related alteration in gait function was observed in 24-month-old C57Bl/6 mice (Tarantini et al. 2018). However, the above-cited studies used a cross-sectional design, that is, each age was represented by a separate group of rats. In the present study, we used a longitudinal design, i.e., the same cohort of animals was followed as they advanced in age. A major difference between the two designs is that, in the former, each rat is exposed to the task for the first time in its life whereas, in our study, aged rats may have relied on their life-long experience and accumulated knowledge when performed the task. Because of this factor, one may expect better performance in a longitudinal than in a cross-sectional design at the same age. More important, the longitudinal design more closely models the human clinical situation, namely, the loss of once already acquired motor skills; thus, its translational value is higher.

The pot jumping task offers a voluntary exercise where performance of animals much depends on their actual motivational state. Thus, the paradigm may sensitively detect changes in motivation. In contrast, tasks like rotarod, treadmill belt, or motorized running wheel and also several motor function assays like wire hanging or tilted plane (Šedý et al. 2008) constitute an “aversive” forced exercise that might even cause anxiety (Leasure and Jones 2008). Deterioration in forced activities may rather occur due to the diminishing physical fitness and emerging movement difficulties. During aging, these changes may evolve to the stage of frailty, a serious clinical syndrome characterized by reduced strength, endurance, speed, and diminished physiologic function (Miller et al. 2017). In the pot jumping paradigm, the increased number of missed jumps and failed overarchings ending in falling/slipping into the water indicated the appearance of frailty what we observed in LE rats from the age of 27 months on. Interestingly, the time-course of this increase (data not shown) completely coincided with the curve of “not leaving” rats. On the other hand, the proportion of jumper rats began to diminish 6 months earlier and reached zero point by the age of 29 months. One may conceive that change in jumping activity reflected the gradual loss of motivation to explore which preceded the development of physical disability signaled by movement faults and reluctance to set off. An example for the assumption that first motivation is lost before physical movement difficulties emerge is the salient point in the curves of longest distance spanned as well as number of “not leaving” and “jumper” LH rats at age of 25 months (Figs. 3 and 6, arrows). At that single occasion, the test was conducted by an intern who was not familiar with the animals. Presumably, the motivation of the animals to inquire the new experimenter might temporary override their general loss of interest so they jumped farther on the pots towards her sitting position close to pot 7 (see Fig. 1).

As recent evidences show a strong correlation between cognitive decline and gait performance in humans (Beauchet et al. 2017; Callisaya et al. 2017; Belghali et al. 2017), the described new method could also be suitable for an early prediction of neurodegenerative changes. For example, in another motor performance model, Tarantini et al. (2017) showed that disrupting neurovascular coupling resulted in an impairment in gait coordination.

Here, we would like to add a note on the transferability of the method to mice, considering that the biogerontological community makes ample use of transgenic mouse models and this is the most widely used non-human species in geroscience. The task may be adapted to mice using proportionally smaller enclosure and jumping surfaces. At the initial phase, mice may require a longer and more gradual habituation to the equipment and testing conditions; however, the exploratory driving force for performing the task should work in this species, too.

In conclusion, the presented newly established “pot jumping” task may be an appropriate and simple model to investigate the development of motor skill learning as well as its age-associated decline or deficits caused by lesions of the motor system. The method seems to be sensitive to detect the beginning and progression of aging process in rats. The described final pot setup could be applied twice a week for young rats from the beginning of training until about 1 year of age, when, usually, their personal best results are achieved. Subsequently, monthly sustaining training could be performed until physical incapability of the animals. More frequent measurements and trials longer than 3 min are not recommended to avoid losing motivation for moving. As and when required for an experimental design of drug testing, tests may be performed more frequently for a certain period. The pot pattern can be optionally varied in order to raise task difficulty. According to the experienced correspondence between rats’ and human age-related behavior, potential enhancer drugs might be tested for improving motor skills and/or procedural memory.

## Electronic supplementary material

ESM 1 (MP4 24,038 kb)

ESM 2 (PDF 11 kb)
