# Supplementary material for: Following of aging process in a new motor skill learning model, “pot jumping” in rats
Source: GeroScience. 2019 May 25;41(3):309–19. doi: 10.1007/s11357-019-00073-3 (PMC6698317; doi:10.1007/s11357-019-00073-3)
Supplement: Supplementary file 2 — (PDF 11 kb) [file 11357_2019_73_MOESM2_ESM.pdf]

journal: Geroscience

Following of aging process in a new motor skill learning model, “pot jumping” in rats

Aliz Judit Ernyey, Tiago Pereira Grohmann, Kata Kozma, Shima Kouhnavardi, Ferenc Kassai, István Gyertyán

MTA-SE NAP B Cognitive Translational Behavioural Pharmacology Group, Department of Pharmacology and Pharmacotherapy, Faculty of Medicine, Semmelweis University, Nagyvárad tér 4, H-1089, Budapest, Hungary

Corresponding author: Aliz Judit Ernyey

Email: ernyey.aliz@med.semmelweis-univ.hu

A sample video is shown on the pot jumping of Lister Hooded rat 5B at age of 13 months. It jumps along until pot 8, then after some hesitation does not jump farther rather returns to the first pot, where it correctly jumps one of the longest distances, 44 cm, and gets the peanut from the paper tube.
